# Supplementary material for: Artemether and Euphorbia factor L9 suppress kynurenine production through distinct effects on tryptophan metabolism
Source: Biochem J. 2026 Apr 2;483(4):541–64. doi: 10.1042/BCJ20253246 (PMC13094657; doi:10.1042/BCJ20253246)
Supplement: Supplementary Figures S1-S7 [file BCJ-2025-3246_supp.pdf]

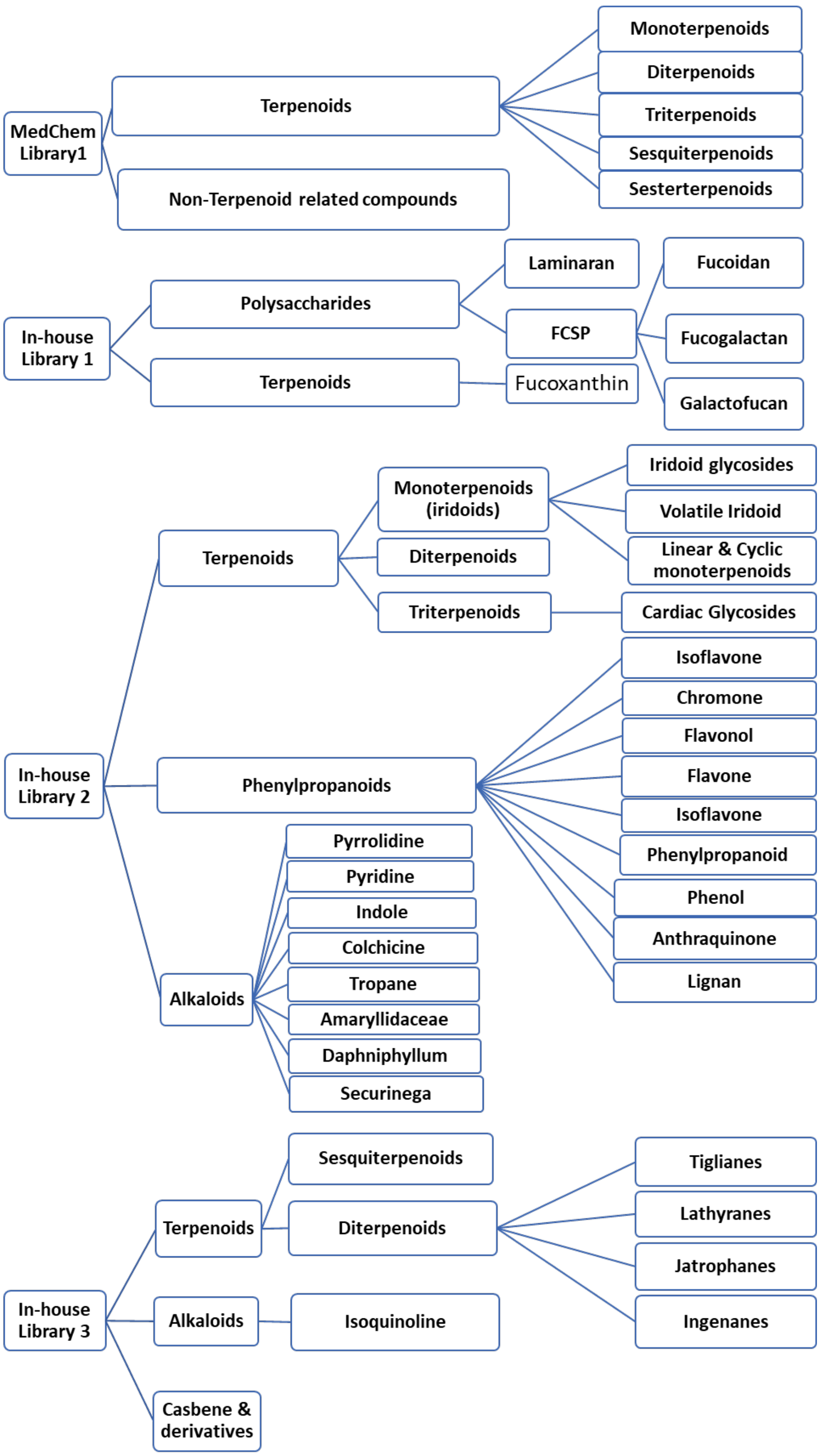

Figure S1. Summary of the main classes of 597 compounds and their sources: commercially available MedChemExpress (MCE) library and three in-house generated libraries. FCSP - Fucose-containing sulphated polysaccharides.

A.

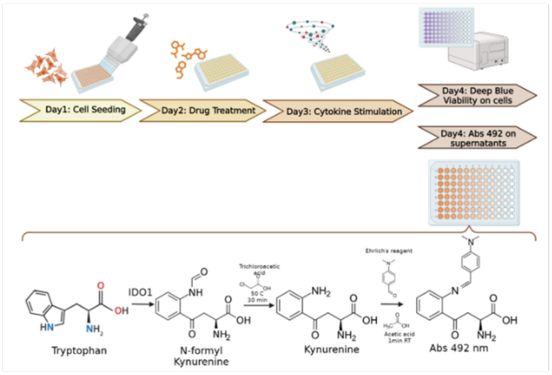

B. **Drug plate**

	1	2	3	4	5	6	7	8	9	10	11	12
A				vehicle unstimulated	1	2	3	vehicle	4	5	6	Ouabain
B				7	8	9	10	11	12	13	14	15
C				16	17	18	19	20	21	22	23	24
D				Ouabain	25	26	27	vehicle unstimulated	28	29	30	vehicle
E				31	32	33	34	35	36	37	38	39
F				40	41	42	43	44	45	46	47	48
G				49	50	51	52	53	54	55	56	57
H				vehicle	58	59	60	Ouabain	61	62	63	vehicle unstimulated

	1	2	3	4	5	6	7	8	9	10	11	12
A	100	100	100	vehicle unstimulated	1	2	3	vehicle	4	5	6	Ouabain
B	50	50	50	7	8	9	10	11	12	13	14	15
C	25	25	25	16	17	18	19	20	21	22	23	24
D	12,5	12,5	12,5	Ouabain	25	26	27	vehicle unstimulated	28	29	30	vehicle
E	6,25	6,25	6,25	31	32	33	34	35	36	37	38	39
F	3,13	3,13	3,13	40	41	42	43	44	45	46	47	48
G	1,56	1,56	1,56	49	50	51	52	53	54	55	56	57
H	0	0	0	vehicle	58	59	60	Ouabain	61	62	63	vehicle unstimulated

Kynurenine standards

	1	2	3	4	5	6	7	8	9	10	11	12
A	100	100	100	vehicle unstimulated	1	2	3	vehicle	4	5	6	Ouabain
B	50	50	50	7	8	9	10	11	12	13	14	15
C	25	25	25	16	17	18	19	20	21	22	23	24
D	12,5	12,5	12,5	Ouabain	25	26	27	vehicle unstimulated	28	29	30	vehicle
E	6,25	6,25	6,25	31	32	33	34	35	36	37	38	39
F	3,13	3,13	3,13	40	41	42	43	44	45	46	47	48
G	1,56	1,56	1,56	49	50	51	52	53	54	55	56	57
H	0	0	0	vehicle	58	59	60	Ouabain	61	62	63	vehicle unstimulated

Kynurenine standards

C.

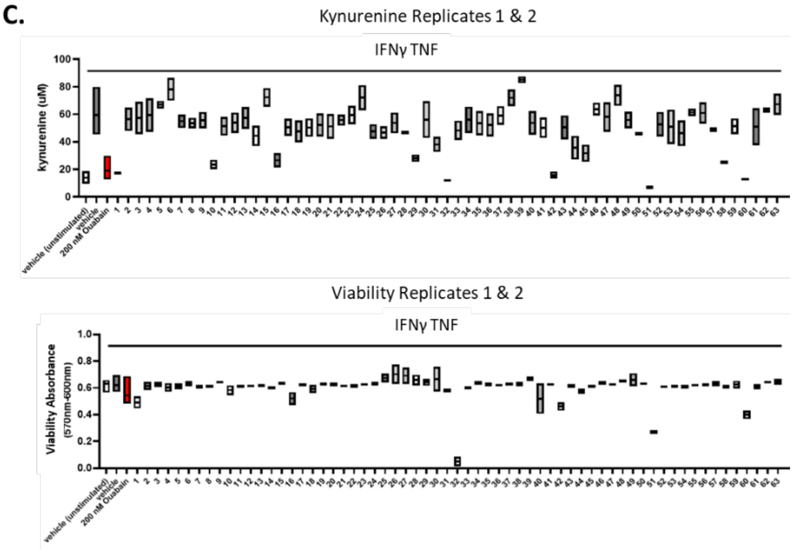

Figure S2. Drug screen set-up and representative raw data per plate. A. Drug screen workflow: the kynurenine assay and deep blue viability; **B.** Drug plate and kynurenine plate maps for each set of compounds; **C.** Representative raw data for the first set of compounds.

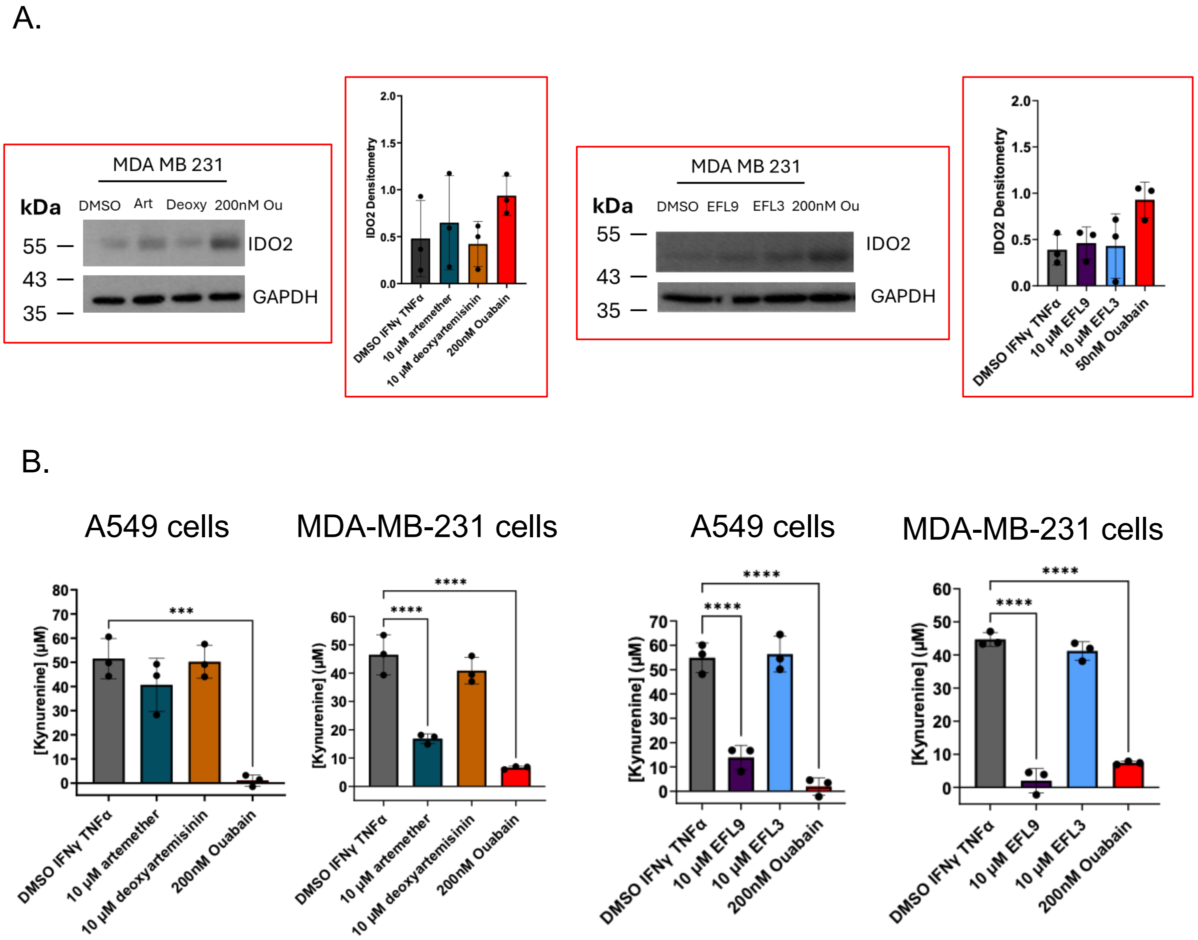

Figure S3. Artemether and EFL9 do not affect IDO2 expression and control kynurenine production in lung cancer cells. A. IDO2 levels in MDA-MB-231 cells following treatment with artemether or deoxyartemether (left) and EFL9 or EFL3 (right). Ouabain was included as a control. Densitometry shown for n=3 independent experiments. **B.** Kynurenine levels (n=3) in IFNγ/TNF-treated A549 lung adenocarcinoma or MDA-MB-231 cells treated with artemether or deoxyartemisinin (left) or EFL9, EFL3 (right). DMSO and ouabain are included as controls. Statistical analysis using ANOVA coupled with a Bonferroni's post-test (*p<0.05, **p<0.01, ***p<0.001, ****p<0.0001).

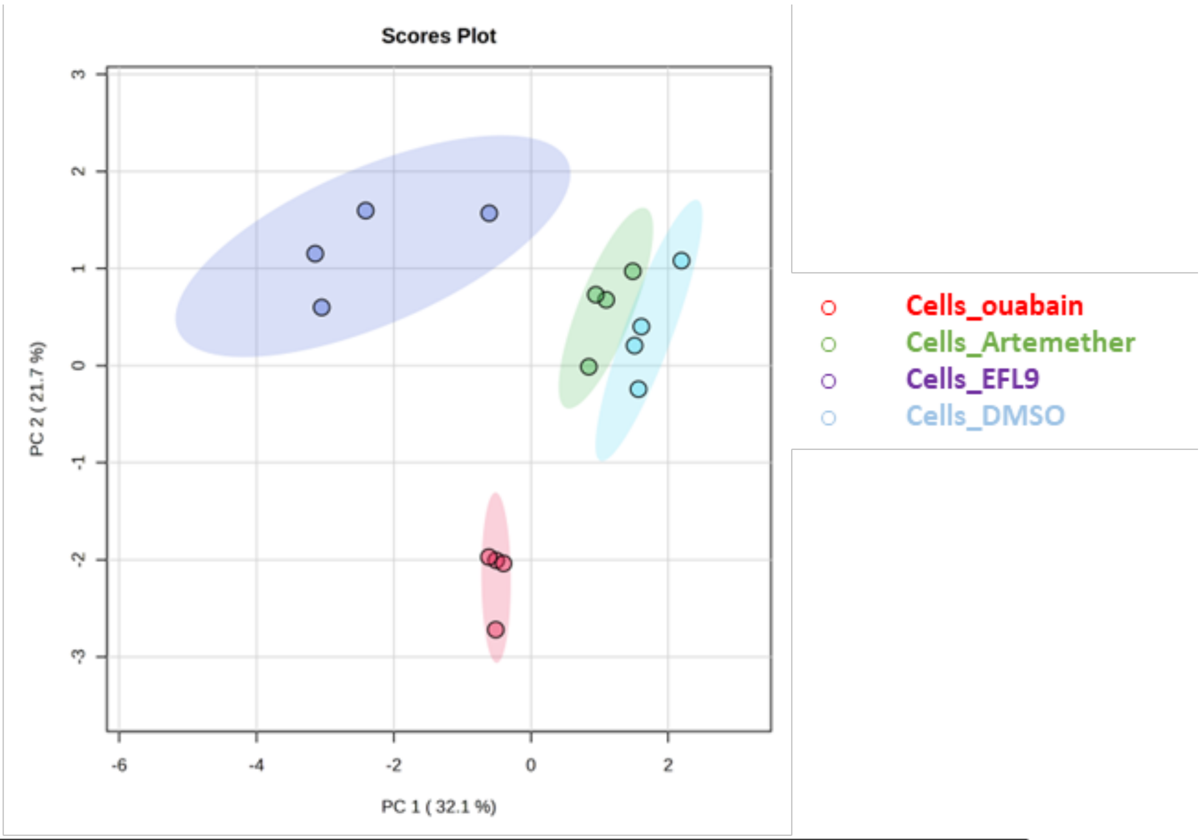

PC1	PC2	PC3	PC4	PC5
32.1%	21.7%	12.1%	9.9%	6.2%

Figure S4. Impact of ouabain, artemether and EFL9 on Trp metabolites in MDA-MB-231 cell extracts. PCA analysis of the entire metabolite output data set (135 metabolites) in cells treated with DMSO (blue), artemether (green), EFL9 (purple), and ouabain (red), n=4 biological replicates.

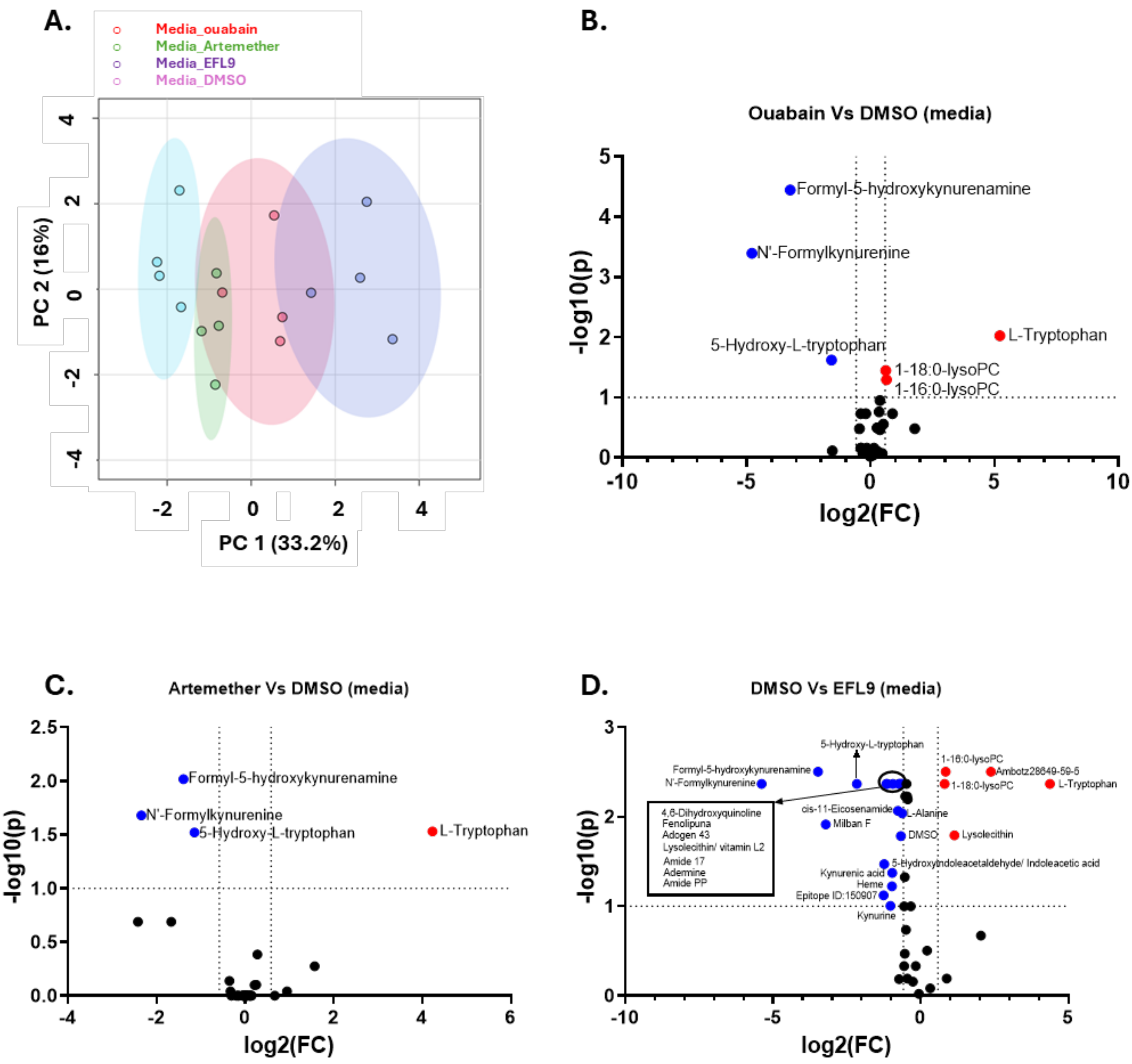

Figure S5. Impact of ouabain, artemether and EFL9 on Trp metabolites secreted by MDA-MB-231 cells. A. PCA analysis of the annotated metabolite data set (135 metabolites with 19 metaboanalyst automatic exclusions), n=4. **B.** Volcano plot of log10(Fold change) Vs log10(p) looking at changes in metabolites in response ouabain, analysis was carried out using the annotated metabolite data set (47 metabolites with 6 metaboanalyst automatic exclusions), n=4. **C.** Volcano plot of log10(Fold change) Vs log10(p) looking at changes in metabolites in response artemether, analysis was carried out using the annotated metabolite data set (47 metabolites with 5 metaboanalyst automatic exclusions), n=4. **D.** Volcano plot of log10(Fold change) Vs log10(p) looking at changes in metabolites in response EFL9, analysis was carried out using the annotated metabolite data set (47 metabolites with 4 metaboanalyst automatic exclusions), n=4.

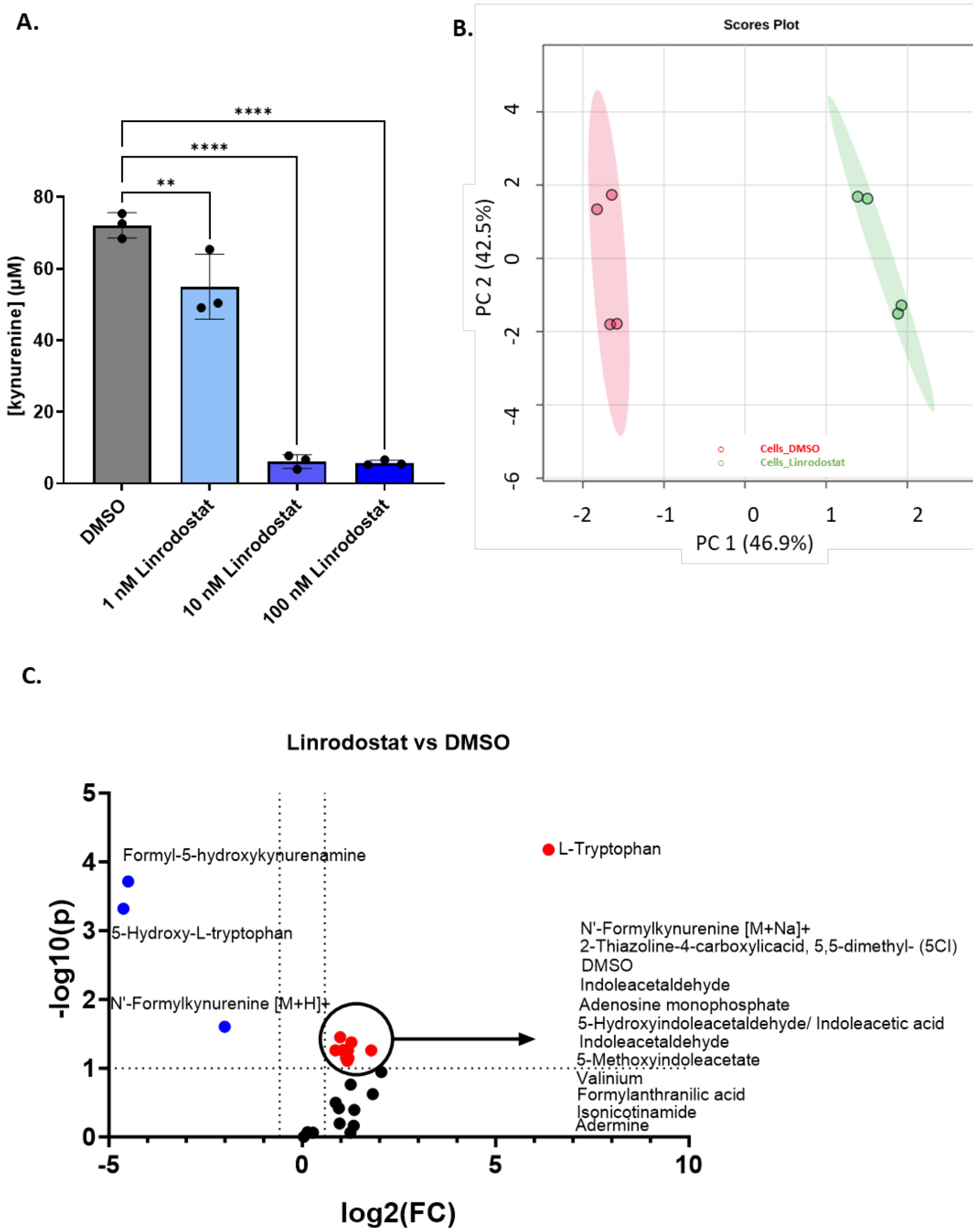

Figure S6. Impact of Linrodostat on Trp metabolism in MDA-MB-231 cells. A. Effect of the commercially available IDO1 inhibitor, Linrodostat, on kynurenine production in MDA-MB-231 cells, n=3 biological replicates. Statistical analysis was carried out using a one-way ANOVA coupled with a Bonferroni's post-test (all conditions were compared to the DMSO control), for specific comparisons (**$p<0.01$; ****$p<0.0001$) **B.** PCA analysis of the annotated metabolite data set (28 metabolites, n=4). **C.** Volcano plot of log10(Fold change) Vs log10(p) looking at changes in metabolites in response Linrodostat, analysis was carried out using the annotated metabolite data set (28 metabolites), n=4.

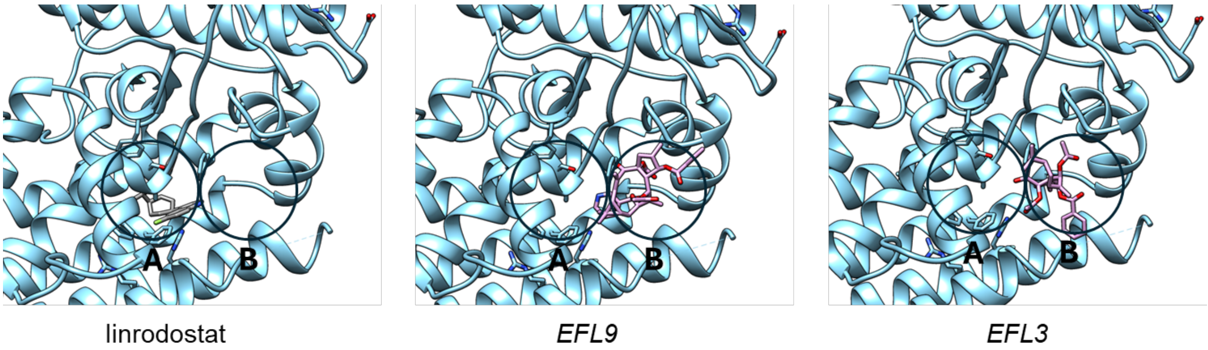

Figure S7. Predicted binding of EFL9 and EFL3 to IDO1 C2 and C3 structures. Binding modes estimated with computational docking. EFL9 and EFL3 are too large to occupy the pocket A in the absence of heme. They remain in pocket B in all binding modes across C2 and C3 structures.

SUPPLEMENTARY TABLES

Supplementary Table 1: Summary of drug screen results and controls
Supplementary Table1.xlsx
Supplementary Table 2: Reported biological activities of compounds of interest (COIs)
Supplementary Table 2.xlsx
Supplementary Table 3: Docking energy values and different poses for EFL9, EFL3, Artmether, and Deoxyartemether
